# Culture, qPCR, and genome-based surveillance of *bla*_*KPC*_, *bla*_*NDM*_, and *bla*_*VIM*_ in school wastewater from Santiago, Chile

**DOI:** 10.3389/fmicb.2026.1787083

**Published:** 2026-04-23

**Authors:** Andrés Cortez-Astorga, Diego Lira-Velásquez, Richard Covarrubia-López, Juan Castro-Severyn, Gabriel I. Krüger, Nicolás Pacheco, Aldo Gaggero, Francisco Remonsellez, Sofía Quintana, Felipe Vásquez-Ponce, Claudia P. Saavedra, Jorge Olivares-Pacheco

**Affiliations:** 1 Grupo de Resistencia Antimicrobiana en Bacterias Patógenas y Ambientales (GRABPA), Instituto de Biología, Pontificia Universidad Católica de Valparaíso, Valparaiso, Chile; 2 Laboratorio de Microbiología Molecular, One Health Institute, Facultad de Ciencias de la Vida, Universidad Andrés Bello, Santiago, Chile; 3 Facultad de Ingeniería y Negocios, Universidad de las Américas, Providencia, Santiago, Chile; 4 Programa de Virología, ICBM, Facultad de Medicina, Universidad de Chile, Santiago, Chile; 5 Laboratorio de Microbiología Aplicada y Extremófilos, Departamento de Ingeniería Química, Universidad Católica del Norte, Antofagasta, Chile

**Keywords:** AMR, *bla*_*KPC*_, *bla*_*NDM*_, *bla*_*VIM*_, carbapenemase genes, *Escherichia coli* ST1193, school wastewater, wastewater-based epidemiology

## Abstract

**Background:**

Antimicrobial resistance (AMR) surveillance remains predominantly centered on clinical settings, potentially underestimating the community circulation of high-priority carbapenemase determinants.

**Methods:**

We integrated culture-based isolation, absolute qPCR, and whole-genome sequencing (WGS) to monitor *bla*_*KPC*_, *bla*_*NDM*_, and *bla*_*VIM*_ in school wastewater from Santiago, Chile. Four 8-h composite influent samples (one per season) were collected in 2024 from an educational establishment comprising primary and secondary levels. Wastewater was screened on MacConkey agar supplemented with ceftazidime or ciprofloxacin (2 μg/mL), followed by disk diffusion testing. Carbapenem-non-susceptible isolates were assessed by Blue-Carba and PCR targeting *bla*_*KPC*_, *bla*_*NDM*_, and *bla*_*VIM*_; carbapenemase-confirmed isolates were further characterized by WGS and resistome analysis. In parallel, gene copy numbers were quantified directly from wastewater DNA using plasmid-based standard curves.

**Results:**

Total bacterial recovery on antibiotic-free control plates was comparable across seasons, supporting consistent sampling performance. Resistance rates peaked in winter for both ceftazidime (3.7% ± 0.87) and ciprofloxacin (11.1% ± 1.72). Across seasons, 92 morphotypes were recovered, dominated by the genera *Aeromonas* (*n* = 43) and *Pseudomonas* (*n* = 17), with *Enterobacterales* most frequent in winter (10/18). Twenty-one isolates were non-susceptible to at least one carbapenem; 12 were Blue-Carba positive (winter, *n* = 9; fall, *n* = 3), while no carbapenem-resistant isolates were recovered in spring and no carbapenemase producers were detected in summer. Multi-carbapenemase genotypes were detected, with *bla*_*NDM*_ restricted to winter. WGS confirmed *bla*_*KPC*_-*bla*_*NDM*_-*bla*_*VIM*_ co-carriage in five winter isolates, including a critical-priority *Escherichia coli* ST1193. Genomes also revealed complex Aeromonas resistomes and the presence of *mcr-1* and *mcr-3.17* in *Aeromonas allosaccharophila* isolates. Absolute qPCR showed a pronounced winter peak (up to 10^7^ copies for *bla*_*KPC*_; >10^6^ for *bla*_*NDM*_ and *bla*_*VIM*_) and low loads in spring-summer, mirroring isolate-based findings.

**Conclusions:**

School wastewater provided a sensitive community sentinel matrix for targeted carbapenemase surveillance, and *bla*_*KPC*_ emerged as a robust biomarker capturing seasonal AMR dynamics.

## Introduction

Antimicrobial resistance (AMR) is among the most pressing threats to global public health, owing to its direct impact on morbidity, mortality, and healthcare costs (Dadgostar, 2019; Murray et al., 2022). The sustained rise of multidrug-resistant bacteria has expanded beyond hospital boundaries and is now established in community settings, further complicating containment (van Duin and Paterson, 2016; Diop et al., 2025). In response, international organizations have promoted diverse surveillance strategies, with the World Health Organization's Global Antimicrobial Resistance System (GLASS) among the most prominent initiatives, primarily focused on hospital-acquired infections caused by priority pathogens (Fang and Schooley, 2020; Marin et al., 2025). However, restricting surveillance to clinical settings underestimates the environmental and community dimensions of AMR, where the circulation of resistant bacteria and antibiotic resistance genes (ARGs) remains largely outside existing surveillance systems, which are currently the dominant and, in some contexts, the only monitoring approach (Hart et al., 2023).

In this context, environmental surveillance has emerged as an essential complementary tool. Among proposed alternatives, wastewater monitoring has consolidated as a promising strategy to assess AMR burden at the population level (Malcom and Bowes, 2025; Foxman et al., 2025). Wastewater can concentrate the three key components relevant to AMR surveillance, namely antibiotic residues (ARs), antibiotic-resistant bacteria (ARB), and ARGs, making it both a reservoir and a critical dissemination point (Malcom and Bowes, 2025; Rosato et al., 2025). These characteristics position wastewater as a comprehensive source of information on the state of AMR in urban communities. Nonetheless, important challenges persist for its implementation as a surveillance tool, including the identification of suitable biomarkers.

Although ARs provide relevant contextual information, measuring ARs in wastewater faces major analytical challenges because many compounds are chemically and biologically unstable and can undergo rapid transformation or degradation in the sewer system and during sampling and storage, complicating accurate quantification in this matrix (Fabregat-Safont et al., 2023; Xu and Kasprzyk-Hordern, 2023; Li et al., 2025). Moreover, detecting ARs does not necessarily imply the presence of resistant bacteria, as residues are often found at subinhibitory concentrations (Larsson and Flach, 2022). In contrast, ARGs and ARBs are regarded as more robust indicators. Carbapenem-resistant bacteria such as *Enterobacterales* and *Acinetobacter baumannii* have been proposed as key ARB because of their clinical relevance, their classification by WHO as critical-priority pathogens, and their frequent detection in wastewater, especially *Enterobacterales* (Mecik et al., 2024; Urzua-Abad et al., 2024; Antochevis et al., 2025). Likewise, genes encoding carbapenemases, which are among the most successful determinants of resistance to carbapenems, including *bla*_*KPC*_, *bla*_*VIM*_, and *bla*_*NDM*_, are considered high-priority molecular biomarkers due to their widespread global distribution and frequent association with highly mobile plasmids that facilitate horizontal spread among bacterial species. These dynamics underpin the classification of carbapenem-resistant bacteria as WHO critical-priority pathogens (Bleichenbacher et al., 2020; Skandalis et al., 2021; Alglave et al., 2025). Collectively, these features make carbapenemase genes strong candidates as AMR markers in complex matrices such as wastewater (Alglave et al., 2025).

Another critical aspect of environmental surveillance is sampling-location selection. While wastewater treatment plants (WWTP) provide a broad overview of AMR in urban areas, the aggregation inherent to WWTP catchments can dilute local signals, particularly in densely populated cities (Huijbers et al., 2023; Punch et al., 2025). This is especially relevant for Santiago, Chile, a city with more than 8 million inhabitants that has been classified by the United Nations as a large city and a potential megacity (cities with >10 million inhabitants) (Montgomery, 2008). Accordingly, sentinel sampling locations representing specific micro-populations have been proposed. Hospitals and educational establishments, given their population density and defined geographic location, offer underexplored potential in this regard.

In parallel, wastewater-based epidemiology (WBE) at the school and building level has emerged as a strategic approach for community public health surveillance, although research in educational settings has predominantly focused on viral monitoring. Extensive evidence exists for SARS-CoV-2 surveillance in schools (Acosta et al., 2025; Solo-Gabriele et al., 2025; Fielding-Miller et al., 2025), including studies that have characterized viral genomic variants (Sachse et al., 2025). School-based WBE frameworks have also been expanded to monitor other high-impact pathogens, including Influenza A (De Melo et al., 2023), enteric viruses (Wolken et al., 2024), and mumps (Joosten et al., 2026). Beyond pathogen detection, wastewater analysis in schools has shown that fecal virome composition is strongly influenced by the age profile of the contributing population (Mejías-Molina et al., 2024). In addition, school-specific WBE has proven useful for toxicological screening, enabling the monitoring of psychoactive substances and the detection of emerging drugs of abuse among student populations (Verovšek et al., 2023). Despite these advances, the application of WBE to study and monitor AMR in educational settings remains remarkably scarce (Hassard et al., 2023; Pico-Tomàs et al., 2025). Notably, longitudinal studies establishing formal AMR surveillance programs in these environments are largely lacking, representing a major knowledge gap in environmental microbiology and public health strategies.

Here, we conducted a 1-year longitudinal analysis of school wastewater in Santiago, Chile, during 2024, integrating culture-based isolation, absolute qPCR quantification of *bla*_*KPC*_, *bla*_*NDM*_, and *bla*_*VIM*_, and whole-genome sequencing of selected carbapenem-resistant-carbapenamse-confirmed isolates. By focusing on these clinically high-priority carbapenemase determinants and combining phenotypic, molecular, and genomic evidence, we evaluated the potential of educational establishments as community sentinel locations for targeted AMR surveillance. Our findings support schools as strategic monitoring points and contribute to emerging efforts to develop decentralized, community-focused surveillance of carbapenemase genes in large urban settings.

## Materials and methods

### Sample collection

Wastewater samples were collected from the influent of the sanitary system at an educational establishment comprising both primary and secondary levels in Santiago, Chile. The school hosts approximately 1,100 students (ages 5–18 years). In 2024, 8-h composite samples (1 L) were collected in May (fall), August (winter), September (spring), and December (summer) (one sample per season) using a portable automated sampler (Teledyne ISCO 6712, Lincoln, NE, USA). Immediately after collection, samples were transferred to autoclaved 1-L high-density polyethylene (HDPE) bottles and transported to the laboratory under a cold chain. Upon arrival, 50 mL of each sample were filtered through 0.22-μm membrane filters (Merck Millipore, Darmstadt, Germany). Each filter was resuspended in 3 mL of 0.85% sterile saline (NaCl) for bacterial isolation and selection. An additional 50 mL aliquot was processed in the same manner for subsequent environmental DNA extraction. Environmental DNA was extracted using the ZymoBIOMICS DNA Miniprep Kit (Zymo Research, Irvine, CA, USA).

### Isolation and selection of resistant bacteria

From the resuspensions obtained from the 0.22-μm filters (Merck Millipore, Darmstadt, Germany), 1:10 serial dilutions were prepared in 0.85% sterile saline, and 100 μL of the 10^−1^−10^−^3 dilutions were plated in triplicate onto MacConkey agar (BD BBL™, Sparks, MD, USA) individually supplemented with ceftazidime (CAZ) or ciprofloxacin (CIP) (Sigma-Aldrich Corporation, St. Louis, MO, USA), each at a final concentration of 2 μg/mL. These conditions were used to isolate multidrug-resistant *Enterobacterales* potentially carrying extended-spectrum β-lactamases (ESBLs) and/or carbapenemases. The antibiotic concentration (2 μg/mL) was selected as a screening concentration to recover bacteria capable of growing under antimicrobial selective pressure while minimizing overgrowth by susceptible flora (Kibwana et al., 2020; Díaz-Gavidia et al., 2021; Araos et al., 2023). A control plate without antibiotics was also included. To prevent yeast overgrowth, cycloheximide was added to the plates at a final concentration of 50 μg/mL Plates were incubated at 37 °C for 24 h. Colony-forming units (CFU) were enumerated at the appropriate dilution and expressed as CFU/mL to estimate the load of resistant bacteria. The resistance rate was calculated as: Resistance rate = (resistant colonies / control colonies) × 100.

Colonies were classified as morphotypes based on colony shape, color, texture, and margin characteristics (Higuera-Llantén et al., 2018). From the antibiotic-supplemented plates, all recognizable morphotypes were collected, and three colonies per morphotype were selected for downstream analyses. To confirm purity, each colony was re-streaked onto the same medium from which it was initially recovered. Pure colonies were then grown overnight at 37 °C in tryptic soy broth (TSB; BD BBL™, Sparks, MD, USA) for DNA extraction using Chelex resin (Bio-Rad, Hercules, CA, USA). Extracted DNA was used as template for 16S rRNA gene amplification.

### Taxonomic identification of isolates

Isolates were identified by 16S rRNA gene sequencing. Total DNA was extracted using Chelex^®^ 100 resin (Bio-Rad, Hercules, CA, USA). The nearly full-length 16S rRNA gene was amplified using GoTaq^®^ Green Master Mix (Promega, Madison, WI, USA) with the universal primers 27F (AGAGTTTGATCCTGGCTCAG) and 1492R (AGRGTTTGATCMTGGCTCAG), following Bertaccini et al. (2019). PCRs were performed in 25 μL reactions containing 50 ng of total DNA (2 μL template). Cycling conditions consisted of an initial denaturation at 95 °C for 5 min, followed by 35 cycles of denaturation at 95 °C for 30 s, annealing at 55 °C for 45 s, and extension at 72 °C for 90 s, with a final extension at 72 °C for 10 min. Amplicons were verified by electrophoresis on 1.0% (w/v) agarose gels stained with SYBR™ Safe DNA Gel Stain (Invitrogen™, Thermo Fisher Scientific, Waltham, MA, USA) and visualized under UV illumination to confirm an expected product of approximately 1,500 bp. Bands were excised and purified using the E.Z.N.A.^®^ Gel Extraction Kit (Omega Bio-tek, Norcross, GA, USA). Purified amplicons were sequenced by Macrogen Inc. (South Korea). Sequences were compared against the EzBioCloud database (www.ezbiocloud.net) to identify the closest taxonomic relatives based on pairwise sequence similarity to reference type strains (Chalita et al., 2024). Isolates were assigned to the genus or species level as permitted by sequence resolution and database match confidence.

### Antimicrobial susceptibility testing

Antimicrobial susceptibility was evaluated using the Kirby–Bauer disk diffusion method on Mueller-Hinton agar (BD BBL™, Sparks, MD, USA), following CLSI guideline M02 (CLSI, 2024a). Briefly, colonies grown for 24 h on tryptic soy agar (BD BBL™, Sparks, MD, USA) were suspended in sterile 0.85% saline and adjusted to a 0.5 McFarland standard. The standardized inoculum was evenly swabbed onto Mueller-Hinton agar, and antimicrobial disks (Oxoid, Basingstoke, United Kingdom) were applied. For each identified morphotype, the following disks (μg) were tested: ampicillin (10), cefazolin (30), cefepime (30), ceftazidime (30), ceftriaxone (30), levofloxacin (5), ciprofloxacin (5), gentamicin (10), amikacin (30), imipenem (10), meropenem (10), azithromycin (15), trimethoprim/sulfamethoxazole (1.25/23.75), piperacillin/tazobactam (100/10), ampicillin/sulbactam (10/10), and aztreonam (30). *Escherichia coli* ATCC 25922 was included as a quality control strain. Plates were incubated at 37 °C for 24 h, after which inhibition zone diameters were measured in millimeters. Zone diameters were interpreted using CLSI clinical breakpoints to classify isolates as susceptible (S), intermediate (I), or resistant (R). *Enterobacterales* were interpreted according to CLSI M100 (CLSI, 2024b), whereas *Aeromonas* spp. were interpreted using CLSI M45 when applicable (CLSI, 2015). For antibiotics not covered by M45, interpretive criteria from M100 were used as a reference, consistent with prior reports (Baron et al., 2017; Wang et al., 2021; Dhanapala et al., 2021).

For infrequently isolated non-*Enterobacterales* taxa with limited CLSI disk diffusion interpretive criteria (*Achromobacter* spp., *Comamonas* spp., and *Pseudomonas* spp. other than *Pseudomonas aeruginosa*), zone diameters were interpreted using CLSI M45 when organism-drug breakpoints were available. When M45 did not provide criteria for a given antibiotic or organism-drug pair, CLSI M100 was used as a reference only when applicable and supported by CLSI-established interpretive criteria (Rong et al., 2022; Harris et al., 2025; Simner et al., 2025).

Isolates were additionally classified as multidrug-resistant (MDR), extensively drug-resistant (XDR), pandrug-resistant (PDR), or non-MDR according to Magiorakos et al. (2012).

### Phenotypic and genotypic detection of carbapenemases

Isolates exhibiting phenotypic non-susceptibility to at least one carbapenem (imipenem and/or meropenem) were further evaluated for carbapenemase production using the Blue-Carba assay, following published procedures (Pires et al., 2013; Pasteran et al., 2015). Briefly, the indicator solution (Solution A) consisted of 0.04% (w/v) bromothymol blue supplemented with ZnSO4 (0.1 mM) and adjusted to pH 7.0. For each isolate, reactions were prepared in 1.5-mL microcentrifuge tubes (Eppendorf, Hamburg, Germany) as paired conditions: a control tube containing 100 μL of Solution A and a test tube containing 100 μL of Solution A supplemented with imipenem (3 mg/mL), prepared fresh immediately before use. Using a sterile loop, five colonies grown on tryptic soy agar (TSA; BD BBL™, Sparks, MD, USA) were suspended in each tube. Tubes were incubated at 35 °C for up to 2 h with periodic mixing, and results were read visually. A test was considered positive when the imipenem-containing tube changed from blue to green/yellow relative to its paired control, consistent with imipenem hydrolysis and a pH shift; reactions were considered negative when no color change was observed within the incubation period. A clinical KPC-producing *E. coli* isolate from our laboratory collection was included as a positive control to verify assay performance.

For molecular confirmation, genomic DNA was extracted from isolates testing positive in the Blue-Carba assay. Briefly, isolates were grown for 24 h at 37 °C in tryptic soy broth (TSB; BD BBL™, Sparks, MD, USA). Cell material was processed using Chelex^®^ 100 resin (Bio-Rad, Hercules, CA, USA) according to the manufacturer's recommendations, and the resulting DNA was used as template for PCR assays. PCR amplification of *bla*_*VIM*_, *bla*_*KPC*_, and *bla*_*NDM*_ was performed using GoTaq^®^ Green Master Mix (Promega, Madison, WI, USA). Primer sequences and cycling conditions are provided in Table 1.

**Table 1:** Primer/probe sequences and PCR/qPCR conditions for *bla*_*KPC*_, *bla*_*NDM*_, and *bla*_*VIM*_.

PCR							
Gene	Primers	Sequence5′ → 3′	Amplicon length (bp)	Assay conditions			References
				T°	Time	Cycles	
***bla**_***VIM***_*			504	95 °C	5′	1	Tiwari et al., 2022
	*bla_*VIM*_* (F)	GGTGTTTGGTCGCATATCGC		95 °C	30″	35	
	*bla_*VIM*_* (R)	CCATTCAGCCAGATCGGCATC			60 °C	15″	
				72 °C	30″		
				72 °C	5′	1	
***bla**_***NDM***_*			452	95 °C	10′	1	Poirel et al., 2011
	*bla_*NDM*_* (F)	GGTTTGGCGATCTGGTTTTC		95 °C	30″	35	
	*bla_*NDM*_* (R)	CGGAATGGCTCATCACGATC		60 °C	15′		
				72 °C	30″		
				72 °C	10′	1	
***bla**_***KPC***_*			798	95 °C	5′	1	Poirel et al., 2011
	*bla_*KPC*_* (F)	CGTCTAGTTCTGCTGTCTTG		95 °C	30″	35	
	*bla_*KPC*_* (R)	CTTGTCATCCTTGTTAGGCG		55 °C	15″		
				72 °C	45″		
				72 °C	5′	1	
**qPCR**							
**Gene**	**Primers**	**Sequence5**′ → **3**′	**Amplicon length (pb)**	Assay conditions			**References**
				**T**°	**Time**	**Cycles**	
***bla**_***KPC***_*	*bla_*KPC*_* (F)	CACTGTGCAGCTCATTCAAG	85	95 °C	3′	1	This study
	*bla_*KPC*_* (R)	ATGGGTGTGTCCAGCAA		95 °C	15″	40	
	*bla_*KPC*_* (P)	CTTTCTTGCTGCCGCTGTGC		60 °C	30″		
***bla**_***VIM***_*	*bla_*NDM*_* (F)	GTTTGGTCGCATATCKCA	87	95 °C	3′	1	This study
	*bla_*NDM*_* (R)	AAGCAACTCATCACCATCAC		95 °C	15″	40	
	*bla_*NDM*_* (P)	CTACCCGTCCAATGGTCTCATT		60 °C	30″		
***bla**_***NDM***_*	*bla_*NDM*_* (F)	CGCGCATCAGGACAAGAT	88	95 °C	3′	1	This study
	*bla_*NDM*_* (R)	GGCAAGCTGGTTCGACAA		95 °C	15″	40	
	*bla_*NDM*_* (P)	CGCATTGGCATAAGTCGCAATCCC		60 °C	30″		

### Whole-genome analysis

Isolates exhibiting phenotypic carbapenem resistance and carrying at least one carbapenemase gene were selected for whole-genome sequencing. Genomic DNA was extracted using the Quick-DNA Miniprep Plus Kit (Zymo Research, Irvine, CA, USA) according to the manufacturer's instructions. Sequencing was performed on the MGI-NSG platform (TCL, Chile).

Bioinformatic analyses included read trimming in Galaxy (Afgan et al., 2022), genome assembly using Unicycler implemented through BV-BRC (Olson et al., 2023), and genome annotation on the same platform. Species assignment was performed using the Type (Strain) Genome Server (TYGS) (Meier-Kolthoff and Göker, 2019), and multilocus sequence types (STs) were determined using PubMLST (Jolley et al., 2018). Antimicrobial resistance genes were identified using ResFinder 4.0 (Bortolaia et al., 2020), PATRIC/BV-BRC (Olson et al., 2023), and the Comprehensive Antibiotic Resistance Database (CARD) (Alcock et al., 2023).

All genome sequences generated in this study have been deposited in NCBI under BioProject accession number PRJNA1395316, and the accession number for each genome is provided in Table 2.

**Table 2:** Whole-genome characterization and resistome profiles of carbapenemase-producing isolates recovered from school wastewater.

MLST	Carbapenamases	ESBL	Resistome	Non-Susceptibility profile	NCBI accession number
*Aeromonas rivipollensis* ST2667	*bla_*OXA*−917_, cphA5, bla_*KPC*_*, *bla_*Vim*_*	*bla_*OXA*−504_*	*aseF, MOX-9, rsmA. CRP*	AMP, KZ, LEV, CIP, IMP, MEM, TZP, SAM	ASM5486433v1
*Aeromonas rivipollensis* ST2466	*cphA5, bla_*KPC*_, bla_*VIM*_*	bla_OXA − 1_, bla_OXA − 504_, bla_OXA − 1041_	*mph(A), aac6′)-Ib-cr, ARR-3, catB3, qacE, sul1, FOX-2*	AMP, KZ, FEP, LEV, CIP, IMP, MEM, AZM, TZP, SAM	ASM5486428v1
*Aeromonas rivipollensis* ST1892	bla_OXA − 917_, bla_KPC_, bla_VIM_	bla_TEM − 156_, bla_OXA − 504_, bla_VEB − 3_	*aac6′)-Ib-cr, mph(A), mph(E), msr(E), qacE, qnrS2, sul1*	AMP, KZ, FEP, CAZ, CRO, LEV, CIP, CN, AK, IMP, MEM, AZM, SXT, TZP, SAM, ATM	ASM5486427v1
*Aeromonas salmonicida* ST1952	*bla_*OXA*−917_, cphA5, bla_*KPC*_, bla_*VIM*_*	*bla_*OXA*−12_*, *FOX-4, bla_*TEM*−156_*	*aac6′)-Ib-cr, aac6′)-Ib-cr, aadA1, aadA16, ARR-3, dfrA27, qacE, qnrVC6, sul1*	AMP, KZ, CAZ, CRO, IMP, TZP, SAM, ATM	ASM5486421v1
*Aeromonas salmonicida* ST1952	*bla_*OXA*−917_, cphA5, bla_*KPC*_, bla_*NDM*_, bla_*VIM*_*	*bla_*OXA*−12_*, *FOX-4, bla_*TEM*−156_*	*aac6′)-Ib-cr, aac6′)-Ib-cr, aadA1, aadA16, ARR-3, dfrA27, qacE, qnrVC6, sul1*	AMP, KZ, FEP, CAZ, CRO, LEV, CIP, IMP, TZP, SAM, ATM	ASM5486425v1
*Aeromonas rivipollensis* ST241	*cphA5, bla_*KPC*_*	*bla_*TEM*−156_*, *bla_*OXA*−504_, mox-9*	*mph(A), aac6′)-Ib-cr, adeF*	KZ, LEV, CIP, AK, IMP, AZM, SXT	ASM5486423v1
*Aeromonas allosaccharophila* ST176	*cphA3, bla_*KPC*_, bla_*NDM*_, bla_*VIM*_*	*bla_*OXA*−1_*, *bla_*TEM*−1*B*_*, *bla_*VEB*−3_*	*aac(3)-IId, aac6′)-Ib-cr, aac6′)- Ib-cr, aadA2, catB3, dfrA12, mcr-1, mcr-3.17, mph(A), qacE, sul1, tet(E)*	AMP, KZ, FEP, CAZ, CRO, CIP, CN, IMP, SXT, SAM	ASM5486417v1
*Aeromonas allosaccharophila* ST2511	*FOX-16, cphA3, bla_*KPC*_, bla_*NDM*_, bla_*VIM*_*	*bla_*OXA*−12_*, *bla_*OXA*−726_*, *bla_*PER*−3_*	*aac6′)-Ib, aac6′)-Ib-cr, mxr, aadA2, catB3, cmlA1, mcr-3.17, mph(A), qacE, sul1, tet(e), FOX-8, aacA16*	AMP, KZ, FEP, CAZ, CRO, CIP, CN, IMP, SAM, ATM	ASM5486431v1
*Escherichia coli* ST1193	*TolC, SoxS, marA, bla_*KPC*_, bla_*NDM*_, bla_*VIM*_*	*bla_*TEM*−1*B*_, evgA*	*mph(A), blaEC-5*	AMP, KZ, LEV, CIP, IMP, AZM	ASM5486419v1

Whole-genome sequencing (WGS) was performed on isolates exhibiting phenotypic non-susceptibility to at least one carbapenem and molecular confirmation of carbapenemase genes (*bla*_*KPC*_, *bla*_*NDM*_, and/or *bla*_*VIM*_). The table summarizes isolate taxonomic assignment and multilocus sequence typing (MLST), together with the resistome detected in each genome, including carbapenemase genes, extended-spectrum β-lactamases (ESBLs), and additional antimicrobial resistance determinants. The detection of clinically relevant genes, including colistin resistance determinants (*mcr* variants), underscores the complexity of resistomes circulating in school wastewater.

### qPCR quantification of *bla_*KPC*_, bla_*VIM*_*, and *bla_*NDM*_*

To minimize biases inherent to culture-based isolation, absolute quantification of *bla*_*KPC*_, *bla*_*VIM*_, and *bla*_*NDM*_ was performed by qPCR. Reactions contained 50 ng of total DNA in 2 μL of template and were prepared using TaqPath™ 1-Step Multiplex Master Mix (Applied Biosystems™, Thermo Fisher Scientific, Waltham, MA, USA). Primer and probe sequences, as well as cycling conditions, are provided in Table 1. Absolute quantification was conducted using gene-specific standard curves, as described below. Each sample was analyzed in triplicate.

### Construction of standard curves for absolute qPCR quantification

For absolute quantification, fragments of *bla*_*KPC*_, *bla*_*VIM*_, and *bla*_*NDM*_ were amplified using the qPCR primers listed in Table 1 and cloned into the TOPO™ TA Cloning™ Kit (Invitrogen™, Thermo Fisher Scientific, Waltham, MA, USA). Plasmid DNA was extracted using the Zyppy™ Plasmid Miniprep Kit (Zymo Research, Irvine, CA, USA) and quantified using a NanoDrop™ spectrophotometer (Thermo Fisher Scientific, Waltham, MA, USA). Plasmid copy number was calculated from the plasmid DNA concentration and the corresponding amplicon length. Ten-fold serial dilutions spanning 10^10^−10^0^ copies were prepared to generate standard curves. All standards were run in triplicate under the same qPCR conditions used for the test samples.

For each target gene, Cq values were plotted against the log10-transformed copy number to generate the standard curve. The slope, coefficient of determination (R^2^), and amplification efficiency were used to assess assay performance. Gene copy numbers in wastewater samples were estimated by interpolation from the corresponding standard-curve equation, as previously described (Seurinck et al., 2005; Olivares and Marshall, 2010; Brankatschk et al., 2012; Churro et al., 2012).

## Results

### Isolation of resistant bacteria by season and resistance rate

Across all sampling dates, bacterial growth was consistently observed on non-antibiotic-supplemented media. Enumeration of colony-forming units (CFU) on antibiotic-free control plates showed that total bacterial recovery remained comparable throughout the year, with values of 5.8 × 106 ± 3.2 × 105 (SD) in fall, 7.3 × 106 ± 2.2 × 104 (SD) in winter, 4.8 × 106 ± 8.1 × 104 (SD) in spring, and 5.7 × 106 ± 3.1 × 105 (SD) in summer. This consistency supports the reliability of the sampling and culture procedures and minimizes potential bias in the estimation of antibiotic resistance rates; accordingly, seasonal differences in resistance are unlikely to be explained by fluctuations in overall bacterial counts.

**Figure 1:**
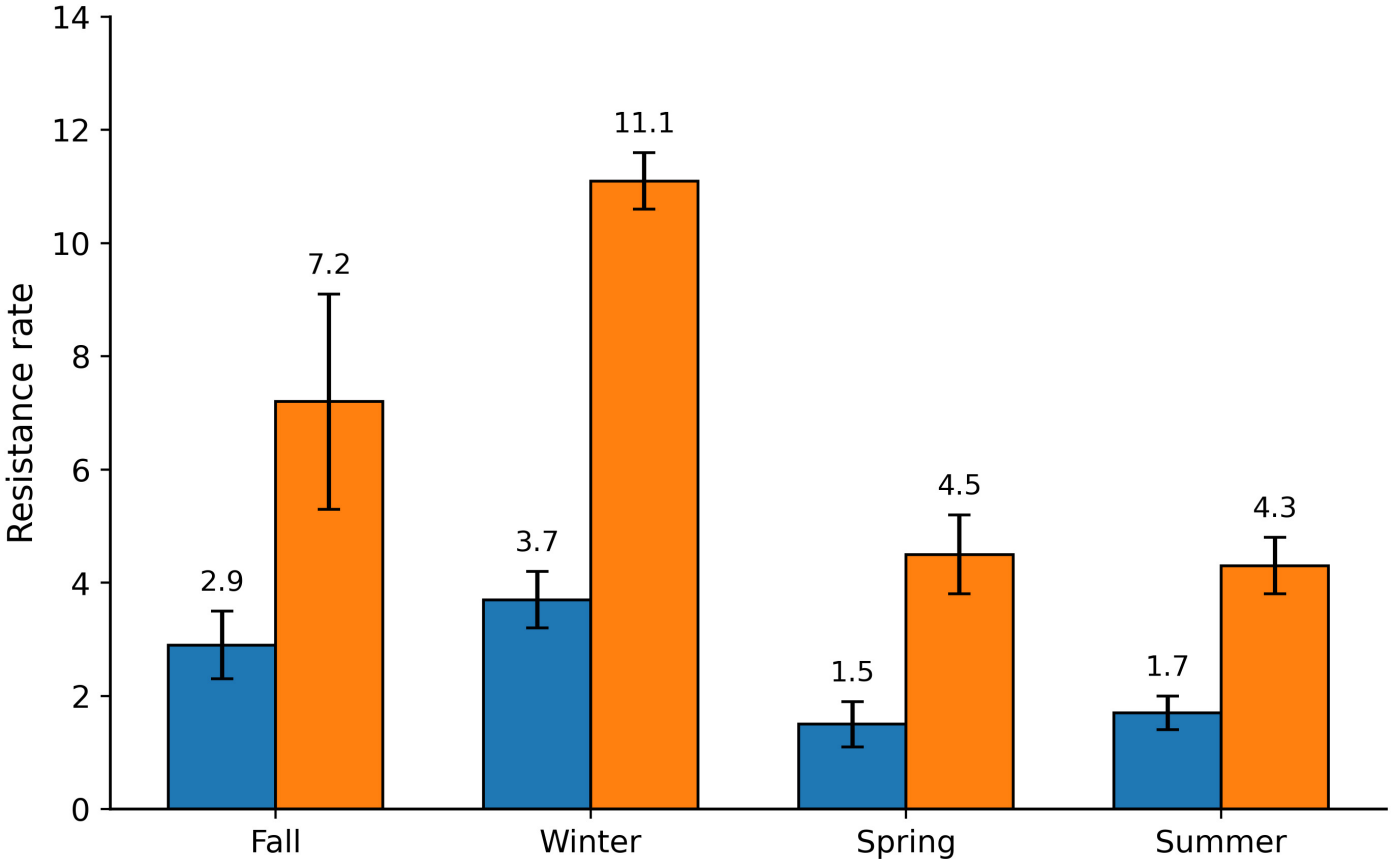
Seasonal variation of resistance rates to ceftazidime and ciprofloxacin. Resistance rates were calculated as the ratio between the number of colony-forming units (CFU) recovered on antibiotic-supplemented plates and those recovered on antibiotic-free plates. Blue bars represent resistance rates to ceftazidime (CAZ), while orange bars represent resistance rates to ciprofloxacin (CIP). Bars show mean values across seasons (fall, winter, spring, and summer), and error bars indicate ± standard deviation.

Resistance rates exhibited clear seasonal variation. Winter showed the highest resistance rates for both ceftazidime and ciprofloxacin (3.7% ± 0.87 and 11.1% ± 1.72, respectively), followed by fall (2.9% ± 0,73 for ceftazidime and 7.2% ± 0.95 for ciprofloxacin) (Figure 1). In spring, ciprofloxacin resistance decreased to 4.5% ± 0.54, while ceftazidime resistance was 1.5% ± 0.32 Summer showed the lowest resistance indices (4.3% ± 1.03 for ciprofloxacin and 1.7% ± 0.21 for ceftazidime) (Figure 1). Overall, winter concentrated the highest isolation of resistant bacteria across the year.

### Taxonomic composition of recovered isolates

Across the four sampling rounds, 92 distinct morphotypes were recovered from CAZ- and CIP-supplemented plates, including 29 in fall, 33 in winter, 14 in spring, and 16 in summer. Based on 16S rRNA gene sequencing, isolates were assigned either to the genus level or, when resolution allowed, to the species level. The collection was dominated by *Aeromonas* (*n* = 43), followed by *Pseudomonas* (*n* = 17). Additional taxa included *E. coli* (*n* = 15), *Comamonas* (*n* = 8), *Haemophilus* (*n* = 4), and *Achromobacter* (*n* = 2), as well as one isolate each classified as *Citrobacter, Hafnia paralvei*, and *Scandinavium geoteborgense* (Table 3). *Aeromonas* predominated in fall and winter, whereas no single genus predominated in spring or summer.

**Table 3:** Taxonomic identification, antimicrobial resistance categories, and carbapenemase detection in bacterial isolates recovered from school wastewater across four seasons.

Season	Species	Resistance profile	Critical priority pathogen	Blue carba	KPC	NDM	VIM
Fall	*Aeromonas rivipollensis*	MDR					
	*Aeromonas salmonicida*	MDR					
	*Aeromonas rivipollensis* ST2667^*^	MDR		+	+		+
	*Aeromonas rivipollensis*	MDR					
	*Haemophilus poscium*	No MDR					
	*Aeromonas rivipollensis* ST2466^*^	MDR		+	+		+
	*Aeromonas salmonicida*	MDR					
	*Haemophilus poscium*	MDR					
	*Aeromonas eucrenophila*	MDR					
	*Aeromonas rivipollensis*	MDR					
	*Pseudomonas alvandae*	MDR					
	*Pseudomonas alvandae*	MDR					
	*Pseudomonas alvandae*	MDR					
	*Aeromonas rivipollensis* ST1892^*^	XDR		+	+		+
	*Pseudomonas siligins*	MDR					
	*Pseudomonas iranensis*	MDR					
	*Pseudomonas jessenii*	MDR					
	*Aeromonas media*	MDR					
	*Aeromonas hidrophila*	MDR					
	*Aeromonas eucrenophila*	No MDR					
	*Aeromonas rivipollensis*	MDR					
	*Escherichia coli*	MDR	+				
	*Aeromonas rivipollensis*	MDR					
	*Haemophilus poscium*	MDR					
	*Aeromonas eucrenophila*	XDR					
	*Aeromonas rivipollensis*	MDR					
	*Escherichia coli*	No MDR					
	*Aeromonas media*	MDR					
	*Aeromonas veronii*	MDR					
Winter	*Aeromonas hidrophila*	MDR					
	*Aeromonas rivipollensis*	MDR					
	*Pseudomonas iranensis*	MDR					
	*Aeromonas media*	MDR		+			
	*Escherichia coli*	MDR					
	*Haemophilus poscium*	MDR					
	*Aeromonas salmonicida* ST1952^*^	MDR		+	+		+
	*Aeromonas salmonicida* ST1952^*^	MDR		+	+	+	+
	*Aeromonas rivipollensis*	MDR					
	*Aeromonas rivipollensis* ST241^*^	MDR		+	+		
	*Aeromonas rivipollensis*	MDR					
	*Aeromonas media*	MDR		+			
	*Aeromonas salmonicida*	No MDR					
	*Pseudomonas piscicola*	MDR					
	*Pseudomonas eucrenophila*	MDR					
	*Escherichia coli*	No MDR					
	*Escherichia coli*	No MDR					
	*Escherichia coli*	MDR	+				
	*Escherichia coli*	MDR	+				
	*Escherichia coli*	MDR	+				
	*Aeromonas cavernicola*	No MDR					
	*Aeromonas allosaccharophila* ST176^*^	XDR		+	+	+	+
	*Aeromonas cavernicola*	MDR					
	*Pseudomonas eucrenophila*	MDR					
	*Aeromonas cavernicola*	MDR					
	*Aeromonas allosaccharophila*	XDR		+			
	*Hafnia paralvei*	No MDR	+				
	*Pseudomonas eucrenophila*	MDR					
	*Aeromonas allosaccharophila*	MDR					
	*Aeromonas allosaccharophila* ST2511^*^	XDR		+	+	+	+
	*Escherichia coli*	No MDR					
	*Escherichia coli* ST1193^*^	MDR	+	+	+	+	+
	*Escherichia coli*	MDR					
Spring	*Escherichia coli*	MDR	+				
	*Pseudomonas defluvii*	MDR					
	*Comamonas jiangduensis*	No MDR					
	*Aeromonas Hydrophila*	XDR					
	*Achromobacter muciolens*	MDR					
	*Comamonas avium*	MDR					
	*Comamonas avium*	No MDR					
	*Comamonas avium*	No MDR					
	*Pseudomonas defluvii*	MDR					
	*Scandinavium goeteborgense*	No MDR	+				
	*Citrobacter freundii*	MDR	+				
	*Aeromonas eucrenophila*	No MDR					
	*Achromobacter piechaudii*	MDR					
	*Pseudomonas defluvii*	MDR					
Summer	*Comamonas jiangduensis*	MDR					
	*Comamonas jiangduensis*	MDR					
	*Pseudomonas defluvii*	MDR					
	*Pseudomonas defluvii*	MDR					
	*Escherichia coli*	MDR					
	*Escherichia coli*	MDR	+				
	*Aeromonas eucrenophila*	MDR					
	*Aeromonas eucrenophila*	MDR					
	*Aeromonas eucrenophila*	MDR					
	*Aeromonas eucrenophila*	MDR					
	*Escherichia coli*	MDR	+				
	*Comamonas avium*	MDR					
	*Pseudomonas defluvii*	MDR					
	*Comamonas jiangduensis*	MDR					
	*Aeromonas rivipollensis*	MDR					
	*Aeromonas rivipollensis*	MDR					

Bacterial isolates recovered from antibiotic-supplemented culture media during fall, winter, spring, and summer were taxonomically assigned and classified by antimicrobial resistance category (MDR, XDR, or non-MDR). Isolates belonging to taxa considered WHO critical-priority pathogens are indicated. Isolates showing phenotypic non-susceptibility to at least one carbapenem were screened for carbapenemase activity using the Blue-Carba assay and subsequently tested by PCR for *bla*_*KPC*_, *bla*_*NDM*_, and *bla*_*VIM*_. Symbols (+) indicate detection of the corresponding gene. Asterisks indicate isolates selected for whole-genome sequencing.

Members of the order *Enterobacterales*, which includes taxa considered potential WHO critical-priority pathogens, accounted for 18 isolates over the study period. Winter yielded the highest number of *Enterobacterales* isolates (*n* = 10), comprising nine *E. coli* and one *H. paralvei* (Table 3). Notably, *A. baumannii*, another WHO critical-priority pathogen, was not isolated at any time point during the study period.

### Seasonal antimicrobial susceptibility patterns

**Figure 2:**
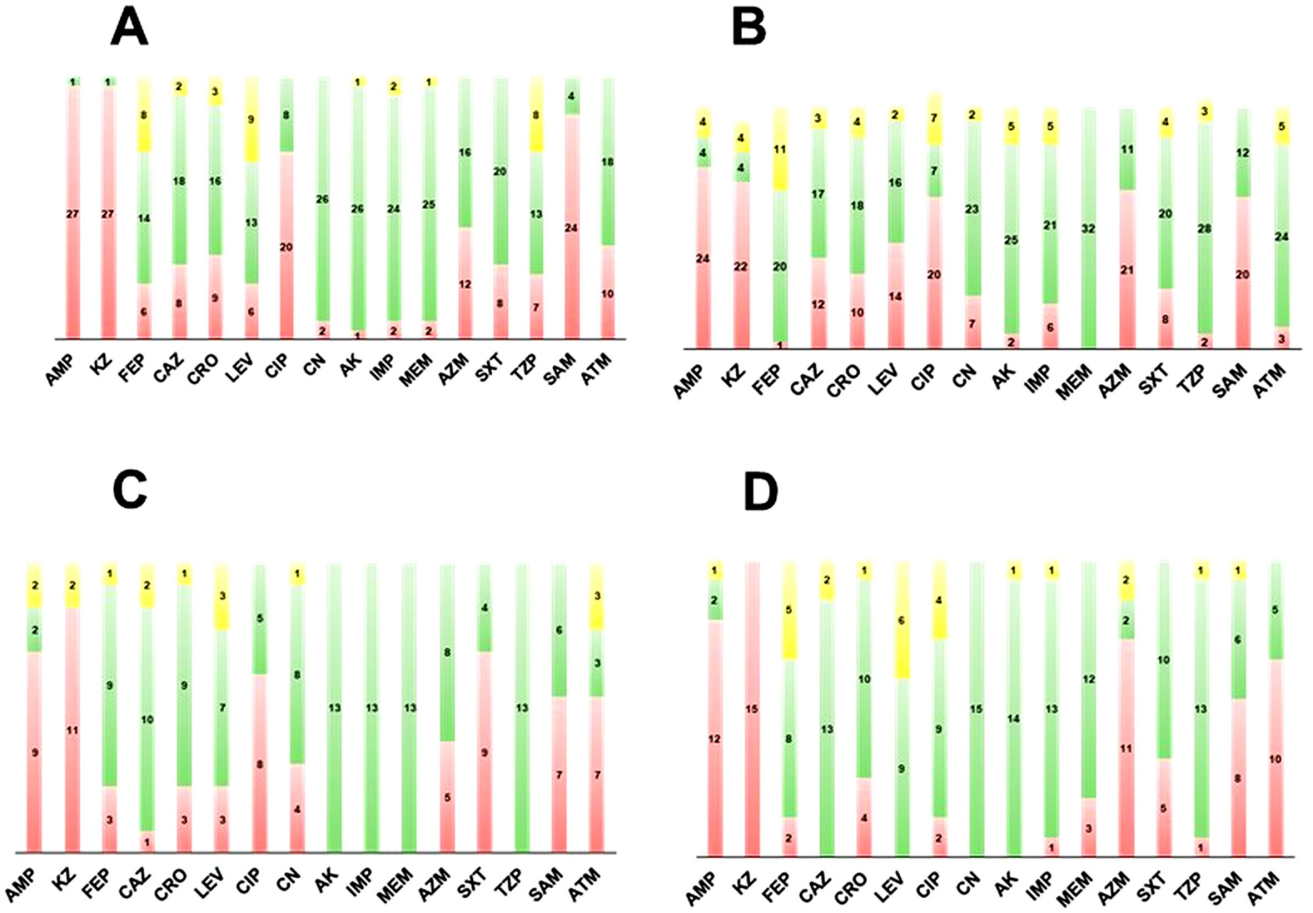
Seasonal distribution of antimicrobial susceptibility profiles of bacterial isolates recovered from school wastewater: Frequency of isolates classified as resistant (red), intermediate (yellow), or susceptible (green) across four seasons: **(A)**: Fall; **(B)**: Winter; **(C)**: Spring; **(D)**: Summer. Each bar represents the number of isolates per antibiotic tested. Antibiotic abbreviations: AMP, ampicillin; KZ, cefazolin; FEP, cefepime; CAZ, ceftazidime; CRO, ceftriaxone; LEV, levofloxacin; CIP, ciprofloxacin; CN, gentamicin; AK, amikacin; IMP, imipenem; MEM, meropenem; AZM, azithromycin; SXT, trimethoprim-sulfamethoxazole; TZP, piperacillin-tazobactam; SAM, ampicillin-sulbactam; ATM, aztreonam.

Seasonal susceptibility profiles are summarized in Figure 2. Across all seasons, resistance to ampicillin and cefazolin remained consistently high. In fall (Figure 2A) and winter (Figure 2B), resistance to ciprofloxacin increased relative to the other sampling periods. For carbapenems, winter yielded the highest number of imipenem-resistant isolates, whereas summer showed the greatest number of meropenem-resistant isolates; summer also exhibited the highest frequency of azithromycin-resistant isolates (Figure 2D).

When analyzed by antibiotic class, cephalosporin non-susceptibility predominated throughout the year. In fall, 100% (29/29) of isolates were non-susceptible to at least one cephalosporin; 48.3% (14/29) were non-susceptible to at least one third-generation cephalosporin, and 31% (9/29) were non-susceptible to both third-generation agents evaluated. In addition, 48.3 (14/29) were non-susceptible to cefepime. In winter, 84.8% (28/33) were non-susceptible to at least one cephalosporin; 45.4% (15/33) were non-susceptible to at least one third-generation agent; and 36.3% (12/33) were non-susceptible to both ceftazidime and ceftriaxone. The same number of winter isolates (12/33) were non-susceptible to cefepime. In spring, cephalosporin non-susceptibility remained universal (100%, 14/14), and 51.7% (8/14) were non-susceptible to at least one third-generation agent; however, no isolate was non-susceptible to both third-generation cephalosporins, and 35.7% (5/14) were resistant to cefepime. In summer, 100% (16/16) of isolates were non-susceptible to at least one cephalosporin; 43.8% (7/16) were non-susceptible to at least one third-generation agent; only one isolate was non-susceptible to both third-generation agents; and 50% (8/16) were non-susceptible to cefepime.

Fluoroquinolone resistance also showed pronounced seasonal variation. Winter exhibited the highest proportion of isolates non-susceptible to at least one fluoroquinolone (75.8%, 25/33) and to both fluoroquinolones tested (48.4%, 16/33). Fall had the highest proportion resistant to both fluoroquinolones (55.2%, 16/29). In spring, more than half of isolates remained non-susceptible to at least one fluoroquinolone (57.1%, 8/14).

Carbapenem non-susceptibility was most evident in winter. Winter showed the highest proportion of isolates non-susceptible to at least one carbapenem (36.3%, 12/33), driven by imipenem non-susceptibility. Summer followed, with 31.3% (5/16) non-susceptible isolates and the highest number of meropenem-resistant isolates (*n* = 3). Fall was the only season with isolates non-susceptible to both carbapenems tested (*n* = 3), whereas no carbapenem-non-susceptible isolates were observed in spring (Figure 2C).

In contrast, aminoglycosides showed the lowest overall frequency of non-susceptibility. Only 23/92 isolates were non-susceptible to at least one aminoglycoside, including 13 in winter, six in spring, three in fall, and one in summer (Figure 2D). Amikacin showed the highest overall activity and accounted for 70% (7/10) of aminoglycoside non-susceptible isolates in winter.

### Isolate-dependent susceptibility patterns across seasons

**Figure 3:**
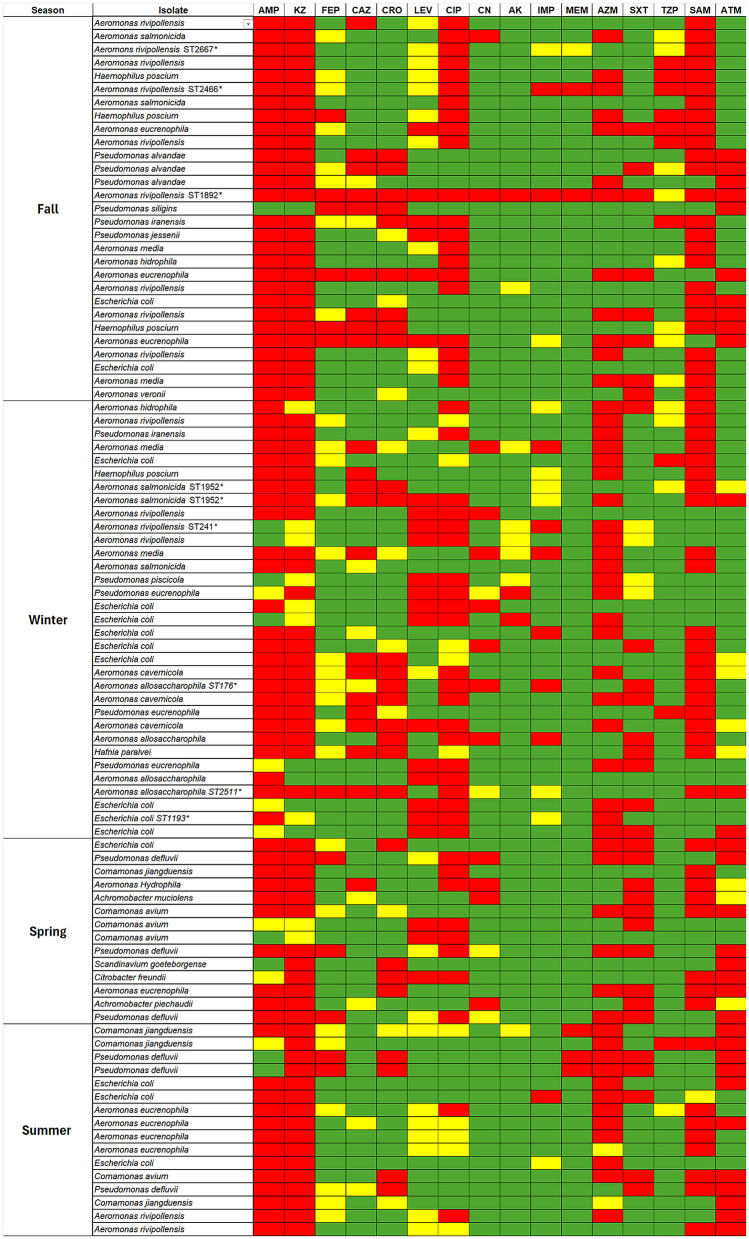
Seasonal antimicrobial susceptibility profiles of bacterial isolates recovered from school wastewater. Heatmap summarizing disk diffusion susceptibility results for isolates recovered from school wastewater across the four sampling rounds (Fall, Winter, Spring, and Summer). Rows correspond to individual isolates, and columns represent the antibiotics tested: ampicillin (AMP), cefazolin (KZ), cefepime (FEP), ceftazidime (CAZ), ceftriaxone (CRO), levofloxacin (LEV), ciprofloxacin (CIP), gentamicin (CN), amikacin (AK), imipenem (IMP), meropenem (MEM), azithromycin (AZM), trimethoprim/sulfamethoxazole (SXT), piperacillin/tazobactam (TZP), ampicillin/sulbactam (SAM), and aztreonam (ATM). Colors indicate categorical interpretations according to CLSI criteria: susceptible (green), intermediate (yellow), and resistant (red). Asterisks indicate isolates selected for whole-genome sequencing.

*Aeromonas* isolates displayed a conserved baseline characterized by non-susceptibility to ampicillin and cefazolin, whereas responses to later-generation cephalosporins, fluoroquinolones, and carbapenems were isolate-dependent. Within *Aeromonas*, more constrained susceptibility profiles clustered in sequence type–resolved lineages, including *A. salmonicida* ST1952 and *A. allosaccharophila* ST176/ST2511. Seasonal differences were also reflected in the distribution of clinically relevant taxa: winter included a higher representation of *Enterobacterales*, particularly *E. coli*, including *E. coli* ST1193, and a subset of these isolates combined non-susceptibility to third-generation cephalosporins and fluoroquinolones. By contrast, spring and summer showed a more mixed genus composition, including *Comamonas* spp. and other less frequent taxa, with more isolate-specific susceptibility patterns across the panel. Across genera, aminoglycosides, especially amikacin, most consistently retained activity, whereas azithromycin showed frequent resistance in this Gram-negative collection (Table 3 and Figure 3).

### Resistance categories and detection of critical-priority pathogens

Isolates were categorized as MDR, XDR, PDR, or non-MDR. Overall, 78.3% (72/92) were classified as MDR, 6.5% (6/92) as XDR, and 15.2% (14/92) as non-MDR; no PDR isolates were detected during the study period.

A total of 11 isolates corresponding to WHO critical-priority pathogens were recovered, including five in winter, three in spring, two in summer, and one in fall (Table 3). These comprised eight *E. coli*, one *H. paralvei*, one *Citrobacter freundii*, and one *S. geoteborgense* (Table 3). Among these, four isolates were non-susceptible to carbapenems (imipenem), and all four were classified as *E. coli*; two were recovered in winter and two in summer. In addition, seven critical-priority isolates were non-susceptible to ceftriaxone, and three were non-susceptible to ceftazidime, with all ceftazidime-non-susceptible isolates recovered in winter. Notably, non-susceptibility to third-generation cephalosporins was observed exclusively in winter, which was also the only season in which an isolate non-susceptible to ceftazidime and resistant to imipenem was identified (Table 3).

### Carbapenemase-producing isolates and carbapenemase gene carriage

Among the 21 isolates that were non-susceptible to at least one carbapenem over the study period, 12 were positive by the Blue-Carba assay. These positives were detected in winter (*n* = 9) and fall (*n* = 3); no carbapenemase producers were detected in summer, and no carbapenem-resistant isolates were recovered in spring. Of the 12 Blue-Carba–positive isolates, 11 belonged to *Aeromonas* and one to *E. coli* (Table 3). PCR-based screening showed that *bla*_*KPC*_ and *bla*_*VIM*_ were the most frequently detected determinants, with nine isolates carrying *bla*_*KPC*_ and *bla*_*VIM*_ (Table 3). In contrast, *bla*_*NDM*_ was detected in five isolates, all recovered in winter. Overall, five winter isolates simultaneously carried *bla*_*KPC*_, *bla*_*VIM*_, and *bla*_*NDM*_, including one *E. coli* classified as a critical-priority pathogen (Table 3). Notably, three Blue-Carba–positive isolates did not amplify any of the carbapenemase genes targeted in this study (*bla*_*KPC*_, *bla*_*VIM*_, *bla*_*NDM*_).

### Whole-genome characterization of carbapenemase-carrying isolates

Whole-genome analysis was performed on isolates exhibiting phenotypic carbapenem resistance and molecular confirmation of carbapenemases, focusing on the clinically and epidemiologically prioritized determinants *bla*_*KPC*_, *bla*_*VIM*_, and *bla*_*NDM*_ (Table 2). MLST derived from whole-genome data identified multiple sequence types among *Aeromonas* isolates, supporting their role as genetically diverse reservoirs of clinically relevant resistance determinants. The two fall isolates (*Aeromonas rivipollensis* ST2667 and *A. rivipollensis* ST2466) carried concurrent *bla*_*KPC*_ and *bla*_*VIM*_, together with the intrinsic Aeromonas-associated carbapenemase *cphA5* (Table 2). These genomes also harbored *bla*_*OXA*_ variants (*bla*_*OXA*−917_, *bla*_*OXA*−504_, and *bla*_*OXA*−12_) and ESBL genes such as *bla*_*TEM*−156_. Although determinants associated with other antibiotic classes (aminoglycosides, phenicols, and sulfonamides) were also detected, the defining feature of this subset was the presence of multiple carbapenemase genes within single genomes (Table 2).

Winter isolates, including *A. rivipollensis* ST1892, *A. salmonicida* ST1952, *A. rivipollensis* ST241, *A. allosaccharophila* ST176, *A. allosaccharophila* ST2511, and E. coli ST1193, exhibited more complex resistomes than fall isolates (Table 2). In addition to *bla*_*KPC*_ and *bla*_*VIM*_, several winter genomes carried *bla*NDM, generating multi-carbapenemase profiles (Table 2). Notably, five winter isolates simultaneously carried *bla*_*KPC*_, *bla*_*VIM*_, and *bla*_*NDM*_, including an *E. coli* isolate classified as a critical-priority pathogen (Table 2). Whole-genome MLST confirmed that this isolate belonged to the epidemic high-risk clone *E. coli* ST1193 (Table 2).

Colistin resistance determinants were detected in *A. allosaccharophila* ST176 and ST2511, including *mcr-1* and *mcr-3.17* (Table 2). The co-occurrence of *mcr* determinants with carbapenemase genes in these isolates further increased the clinical relevance of the resistomes recovered from school wastewater (Table 2). Collectively, these genomic data indicate that carbapenemase-carrying isolates in this setting frequently harbored high-concern resistance configurations, including multi-carbapenemase genotypes and, in a subset, colistin resistance determinants.

### Absolute qPCR quantification of *bla_*KPC*_, bla_*NDM*_*, and *bla_*VIM*_* in wastewater

To complement culture-based analyses and reduce bias associated with cultivable bacteria, absolute qPCR quantification of *bla*_*KPC*_, *bla*_*NDM*_, and *bla*_*VIM*_ was performed directly on wastewater DNA. Standard curves were generated for each target gene, and the corresponding performance metrics are reported in the Supplementary material 1. Amplification efficiencies exceeded 90% for all targets, and the coefficients of determination (R^2^) were 0.995 for *bla*_*KPC*_, 0.991 for *bla*_*VIM*_, and 0.995 for *bla*_*NDM*_, supporting the use of these curves for copy-number estimation by interpolation.

**Figure 4:**
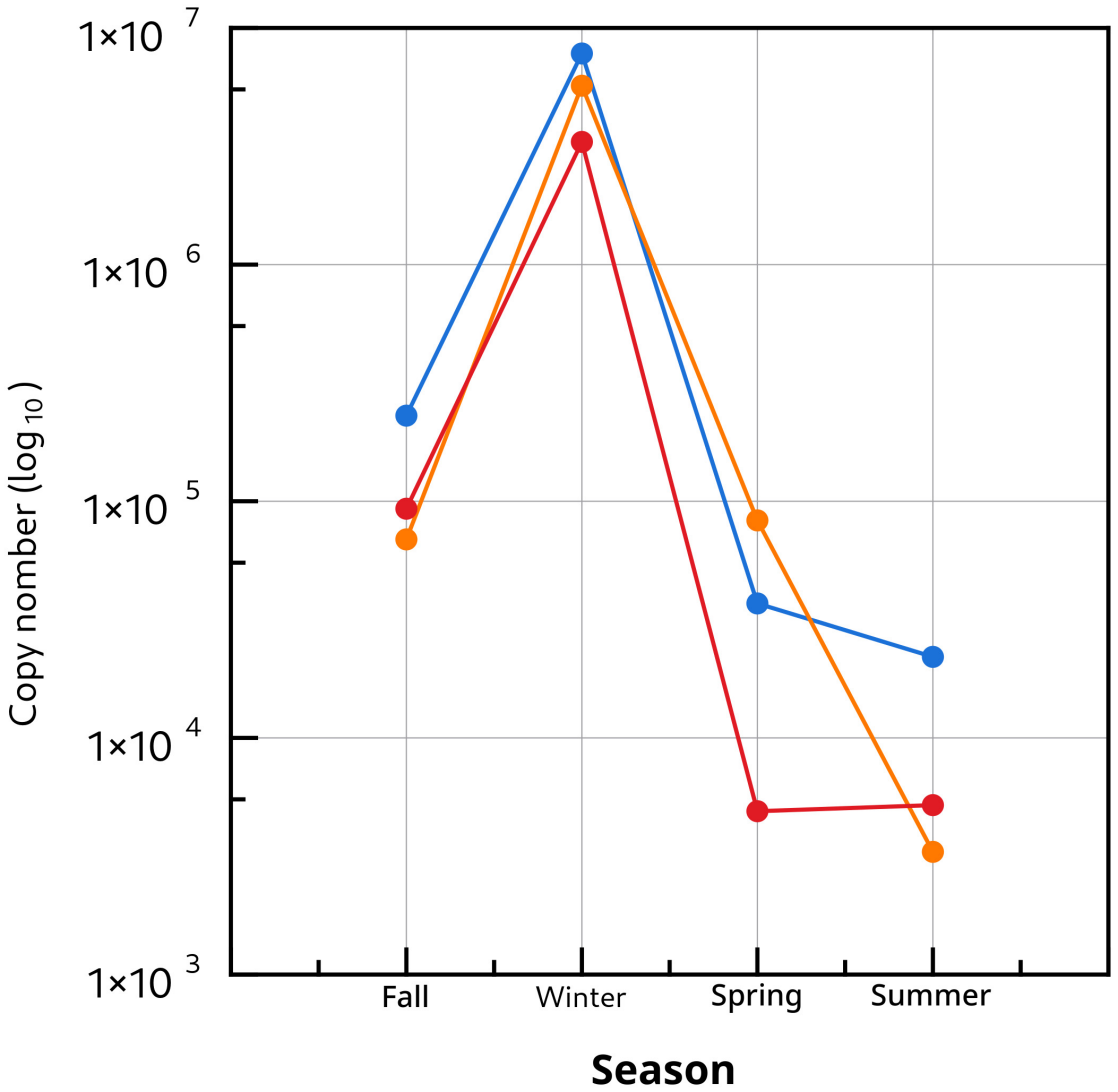
Seasonal absolute quantification of carbapenemase genes in school wastewater: Absolute copy numbers (log10 scale) of *bla*_*KPC*_ (blue), *bla*_*NDM*_ (red), and *bla*_*VIM*_ (orange) quantified by qPCR across four seasons (Fall, Winter, Spring, and Summer). The data show a marked increase in all three carbapenemase genes during winter, with *bla*_*KPC*_ reaching the highest abundance, followed by *bla*_*VIM*_ and *bla*_*NDM*_, and a sharp decline in spring and summer.

qPCR quantification revealed a seasonal pattern that closely aligned with isolate-based observations (Figure 4). In fall, all three carbapenemase genes were detected at approximately 105−106 copies, with *bla*_*KPC*_ as the most abundant target. Winter showed the strongest increase, reaching up to 107 copies for *bla*_*KPC*_, followed by *bla*_*NDM*_ and *bla*_*VIM*_ at >106 copies. This peak coincided with the season showing the highest frequency of carbapenem-resistant isolates and the highest occurrence of multi-carbapenemase genotypes, including triple-positive isolates (*bla*_*KPC*_, *bla*_*NDM*_, *bla*_*VIM*_). In spring, gene loads decreased sharply, particularly for *bla*_*VIM*_ (approximately 103 copies), consistent with the absence of carbapenem-resistant isolates in that season. Summer exhibited the lowest levels for all three genes (103-104 copies), in line with the lowest proportion of non-susceptible isolates.

Overall, winter represented the period of greatest carbapenemase circulation at both phenotypic and molecular levels, with a clear predominance of *bla*_*KPC*_. Across the study period, *bla*_*KPC*_ was consistently the most abundant target and best reflected seasonal resistance trends observed among recovered isolates, supporting its use as a priority sentinel biomarker for community AMR surveillance in school wastewater (Table 3).

## Discussion

This study demonstrates that school wastewater can function as a highly informative sentinel matrix for community antimicrobial resistance surveillance. By integrating culture-based isolation, qPCR quantification of high-priority resistance genes, and whole-genome sequencing of selected carbapenem-resistant, carbapenemase-confirmed isolates, our approach captures both phenotypic resistance in recoverable antibiotic-resistant bacteria and matrix-level burdens of *bla*_*KPC*_, *bla*_*NDM*_, and *bla*_*VIM*_. This multi-layer design strengthens inference beyond what is possible from cultivable bacteria alone and reinforces the public health relevance of environmental AMR surveillance as a complement to clinical systems, particularly for detecting and contextualizing resistance determinants circulating outside healthcare settings.

A central implication of our data is the consistent seasonal structure observed across phenotypic and molecular layers. Winter concentrated the highest frequency of resistant isolates and the greatest loads of carbapenemase genes, particularly *bla*_*KPC*_, followed by *bla*_*NDM*_ and *bla*_*VIM*_. This temporal pattern is epidemiologically meaningful because winter months are associated with increased antimicrobial use and intensified selective pressure in the community (Rawat and Nair, 2010; Codjoe and Donkor, 2017). The seasonal signal also aligns with national observations reported by the Instituto de Salud Pública de Chile, indicating increased reports of carbapenemase-producing *Enterobacterales* during winter, including isolates with double and even triple carbapenemase carriage (Hormazábal, 2023). The concordance between isolate recovery and wastewater gene-load patterns supports *bla*_*KPC*_ as a high-priority sentinel biomarker for community-facing wastewater surveillance.

Beyond matrix-level signals, isolate recovery provides an additional public health dimension by identifying potential bacterial hosts and ecological reservoirs. While a substantial fraction of isolates corresponded to environmental taxa such as *Aeromonas*, which are increasingly recognized as reservoirs of clinically relevant ARGs (McLellan and Roguet, 2019; Milligan et al., 2023), we also recovered a clinically important lineage, *E. coli* ST1193, a globally disseminated extraintestinal pathogenic clone associated with urinary tract and bloodstream infections (Pitout et al., 2022). Importantly, whole-genome sequencing demonstrated that the ST1193 isolate harbored *bla*_*KPC*_, *bla*_*NDM*_, and *bla*_*VIM*_. The detection of this high-risk lineage carrying multiple carbapenemase determinants in school wastewater underscores the epidemiological value of educational settings as sentinel sites and supports the broader interpretation that critical resistance configurations circulate in the community beyond clinical boundaries. Crucially, the ST1193 finding was not an isolated event but part of a broader pattern with direct implications for dissemination risk. Across the sequenced subset, multi-carbapenemase carriage was recurrent, including co-carriage of *bla*_*KPC*_ and *bla*_*VIM*_ in multiple *Aeromonas* genomes, together with the intrinsic Aeromonas-associated carbapenemase (*cphA5*) and additional beta-lactamase determinants (e.g., *bla*_*OXA*_ variants and ESBLs), reinforcing *Aeromonas* as a diverse reservoir of clinically relevant ARGs (McLellan and Roguet, 2019; Milligan et al., 2023). Even more concerning, winter yielded isolates with higher complexity resistomes, including strains carrying *bla*_*NDM*_ in addition to *bla*_*KPC*_ and *bla*_*VIM*_; notably, five winter isolates simultaneously carried all three carbapenemase genes (*bla*_*KPC*_, *bla*_*VIM*_, and *bla*_*NDM*_). From a surveillance perspective, this indicates not only the circulation of individual carbapenemases but also the emergence of convergent, high-concern genotypes at the community level, configurations that can reduce therapeutic options and may facilitate persistence under diverse antibiotic exposures.

The detection of colistin resistance determinants further elevates the public health implications. We identified *mcr-1* and *mcr-3.17* in *A. allosaccharophila* isolates. The presence of *mcr-1* is particularly relevant because it compromises a last-resort antimicrobial and has frequently been associated with mobile genetic elements (Gonzalez-Avila et al., 2021). Most importantly, *mcr-1* co-occurred with carbapenemase determinants within single isolates, reinforcing concern about the potential emergence and maintenance of highly drug-resistant profiles in environmental reservoirs. While our findings establish co-carriage within isolates, the extent to which these determinants are physically co-localized on the same mobile element (vs. distributed across distinct replicons or genomic regions) remains unresolved and is central for understanding co-selection and transmission dynamics.

Addressing this gap does not rely exclusively on long-read sequencing. Although long-read approaches (e.g., Oxford Nanopore) are ideal for resolving plasmid structures and definitively establishing co-localization, short-read sequencing can still support plasmid replicon detection and contig-level inference of gene neighborhoods, and *in silico* approaches may delineate gene locations in some cases. Where genomic resolution remains limited, or where genetic data are unavailable, conjugation (mating) assays and/or transformation experiments can complement sequencing-based analyses to assess transferability and strengthen inference on plasmid-mediated dissemination. Integrating these approaches is particularly important here because multi-carbapenemase and mcr-mediated profiles increase the potential consequences of horizontal spread in community-associated environments (Gonzalez-Avila et al., 2021).

Taken together, our results suggest that resistance signals in school wastewater are shaped by seasonal and environmental factors and plausibly amplified by increased antibiotic exposure during winter months (Rawat and Nair, 2010; Codjoe and Donkor, 2017; Hormazábal, 2023). The repeated detection of multi-carbapenemase carriage, including triple-carbapenemase genotypes, the recovery of a high-risk *E. coli* ST1193 carrying *bla*_*KPC*_, *bla*_*NDM*_, and *bla*_*VIM*_ (Pitout et al., 2022), and the identification of *mcr* determinants (Gonzalez-Avila et al., 2021) underscore the urgency of incorporating community environments, such as schools, into AMR surveillance frameworks. Extending sampling to all months of the year and replicating this approach across schools in different regions will help consolidate educational institutions as scalable community sentinel sites for tracking high-concern resistance determinants and their convergence in bacterial hosts.

## Data Availability

The datasets presented in this study can be found in online repositories. The names of the repository/repositories and accession number(s) can be found below: https://www.ncbi.nlm.nih.gov/bioproject/PRJNA1395316/, PRJNA 1395316.
