# Supplementary material for: Culture, qPCR, and genome-based surveillance of blaKPC, blaNDM, and blaVIM in school wastewater from Santiago, Chile
Source: Front Microbiol. 2026 Apr 23;17:1787083. doi: 10.3389/fmicb.2026.1787083 (PMC13149482; doi:10.3389/fmicb.2026.1787083)

Supplementary material

Supplementary Figure 1. Standard curve for absolute qPCR quantification of *bla_{KPC}* generated from a TOPO™ TA-cloned plasmid standard. The *bla_{KPC}* qPCR amplicon was cloned into the TOPO™ TA vector and used to prepare ten-fold serial dilutions to construct the calibration curve. Cq values were plotted against the log10-transformed copy number to derive the standard-curve equation used for sample copy-number interpolation.

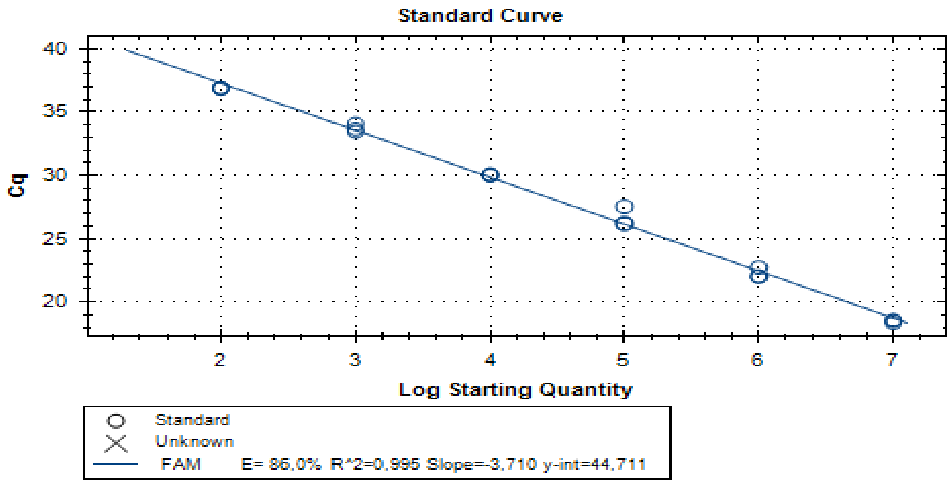

Supplementary Figure 2. Standard curve for absolute qPCR quantification of *bla$_{VIM}$* generated from a TOPO™ TA–cloned plasmid standard. The *bla$_{VIM}$* qPCR amplicon was cloned into the TOPO™ TA vector and used to prepare ten-fold serial dilutions to construct the calibration curve. Cq values were plotted against the log10-transformed copy number to derive the standard-curve equation used for sample copy-number interpolation.

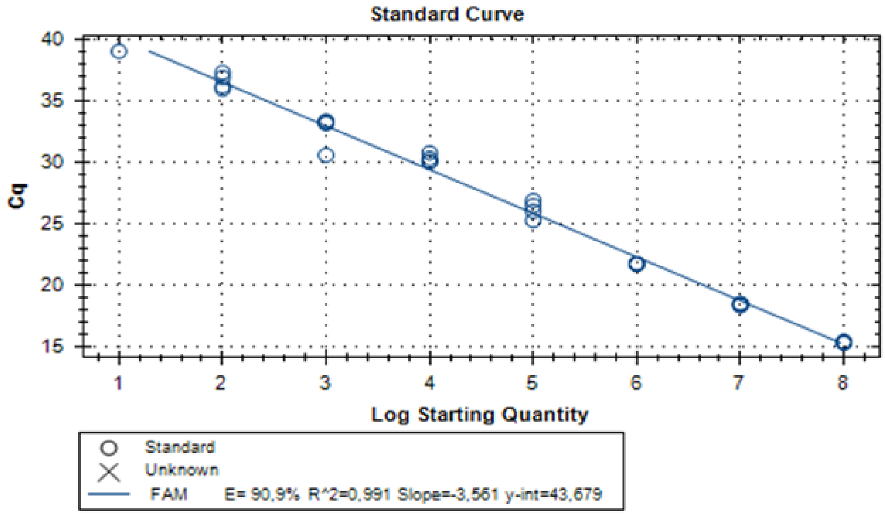

Supplementary Figure 3. Standard curve for absolute qPCR quantification of *bla_{NDM}* generated from a TOPO™ TA–cloned plasmid standard. The *bla_{NDM}* qPCR amplicon was cloned into the TOPO™ TA vector and used to prepare ten-fold serial dilutions to construct the calibration curve. Cq values were plotted against the log10-transformed copy number to derive the standard-curve equation used for sample copy-number interpolation.

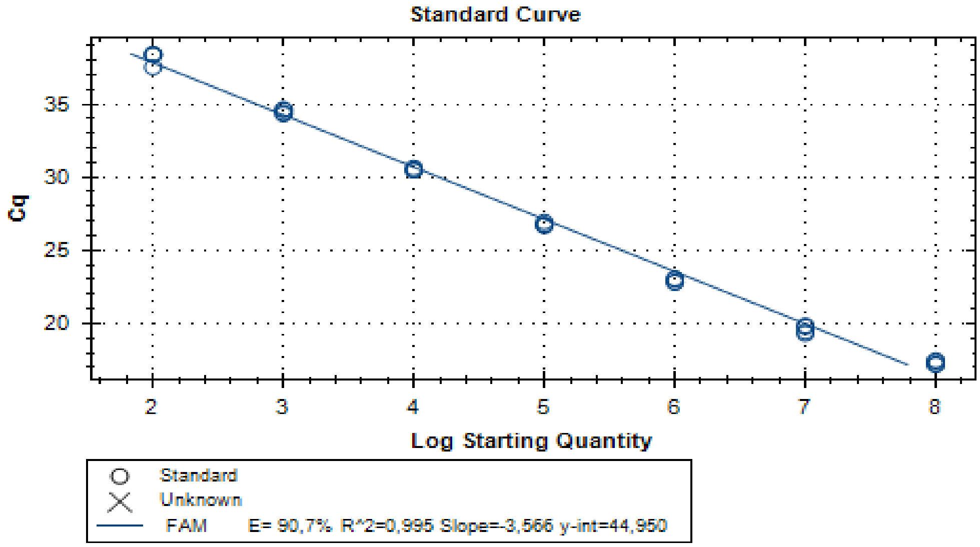

Supplement: Supplementary file 1 [file Data_Sheet_1.pdf]
